# Challenges of Dementia Care in China

**DOI:** 10.3390/geriatrics2010007

**Published:** 2017-01-18

**Authors:** Zheng Chen, Xuan Yang, Yuetao Song, Binbin Song, Yi Zhang, Jiawen Liu, Qing Wang, Jia Yu

**Affiliations:** Institute for Geriatrics and Rehabilitation, Beijing Geriatric Hospital, Beijing University of Chinese Medicine, 118 Wenquan Road, Haidian District, 100095 Beijing, China; paul_c99@sina.com (Z.C.); xuanyang@mail.ccmu.edu.cn (X.Y.); syt521800@sohu.com (Y.S.); sbinbin2012@163.com (B.S.); lnyykjc@163.com (Y.Z.); no818@163.com (J.L.); bghigr@163.com (Q.W.)

**Keywords:** dementia, care, China, public health

## Abstract

Dementia results in brain dysfunction, disability and dependency among affected people, causing an overwhelming burden for caregivers. China has the largest number of people with dementia worldwide and is facing severe challenges with respect to dementia care, including poor awareness of dementia in the public, inadequate knowledge of dementia for medical professionals and caregivers, an underdeveloped dementia service system, and high costs of dementia care. To address these challenges, China is taking action to increase dementia awareness and education among the public and care providers, and develop policies, services and resources for dementia care.

## 1. Introduction

Dementia is a common syndrome among older people, characterized by progressive deterioration of cognitive function, psychological behavior and ability to manage daily life. Although increased risk of dementia is associated with age, dementia is not a normal part of aging [1]. Dementia is divided into several types including Alzheimer's disease (AD), vascular dementia (VaD), dementia with Lewy bodies (DLB) and frontotemporal dementia (FTD). AD is the most common type of dementia, contributing to 50%–75% of dementia cases. VaD, FTD and DLB account for 20%–30%, 5%–10%, and less than 5% of dementia cases, respectively [2]. The process of dementia is irreversible. People with dementia will eventually lose the ability to manage daily life and have to depend on the care of others at the late stage of disease.

Dementia exerts profound impacts on affected individuals, their caregivers and society as a whole. For people with dementia, the disease leads to cognitive dysfunction, behavioral and psychological complications and increased incidence of other chronic comorbidities, such as delirium, falls, urinary incontinence, fractures and infections [3]. For caregivers, dementia causes stress, anxiety, depression, isolation, sleep problems, chronic fatigue, and increased consumption of psychotropic drugs [4]. For society, dementia puts great pressure on the health care system and economic development [5].

## 2. Dementia Prevalence in China

Globally, 47.5 million people have dementia and there are 7.7 million new cases every year [1,6]. China ranks first in the world in terms of the number of people with dementia, accounting for 20% of dementia cases worldwide [7]. The Law of the People’s Republic of China on the Protection of the Rights and Interests of the Elderly defines those belonging to the age group of 60 years and above as older people. According to the Statistical Bulletin for the Development of Social Services in 2015 by the Chinese government, at the end of 2015, the population of older people in China was 222 million, of which 9.5 million were living with dementia.

The incidence or prevalence of dementia shows social-demographic differences. A meta-analysis in 2013 revealed that the overall estimate of dementia prevalence in mainland China was 4.6% in the population aged 60 and over [8]. A survey conducted by China Elderly Healthcare Association showed that the incidence of dementia increased by 1.4% every year in Shanghai and other developed cities [9]. Another study showed that the prevalence of dementia reached 5.00%–7.69% among people over 65 years old, and steeply increased to more than 40% among those over 85 years old [7]. Gender seems to be an important factor that influences the dementia prevalence. According to a report, the incidence ratio of AD was 7:13 between males and females [10]. A study found a cohort effect on dementia prevalence might be due to malnutrition in childhood and the lack of education in early life [11]. However, another study failed to prove the association between nutrition and prevalence of dementia in mainland China, possibly because older people did not experience dietary changes in early to middle life [12]. It should be noted that methodological factors (diagnostic criteria, whole study age range, sampling method and study size), socioeconomic level or life expectancy among areas might influence the accuracy of the estimates of prevalence, incidence of dementia and total number of people with dementia in China [8].

There are regional differences in the incidence or prevalence of dementia. It was estimated that the prevalence of dementia among people over 60 years old was 5.1%, 4.4% and 3.9% in the northern, southern and central regions of China, respectively [8]. High prevalence of dementia was found to be concentrated in metropolitan cities and east-coastal provinces which had greater economic development and political status [8]. The Provisional Regulations of the People’s Republic of China on Statistic Delimitation of Urban and Rural Area define cities and towns as urban areas which occupy 85,800 square kilometers, accounting for only 0.9% of total area of China. All the remaining regions are defined as rural areas (or villages). Normally, a city or town in China has more than 3000 residents, while a village has less than 3000 residents. Among different types of dementia, the prevalence of AD and VaD is 5.9% and 1.3% among people over 65 years old [9]. It was reported that rural areas had a higher incidence of AD, while urban areas had higher incidence of VaD [10]. A study raised the concern that there might be systematic under-ascertainment in rural sites because of low levels of education and awareness, under reporting and under-detection of dementia [13].

## 3. Current Challenges and Issues of Dementia Care in China

Dementia-related issues have gained increasing attention and concern from the Chinese government, policy makers, experts and scholars. However, the public, the medical professionals and caregivers, the health service and insurance system in China are not prepared to face the challenges of dementia care.

### 3.1. Poor Awareness of Dementia in the Public

In 2012, the World Health Organization (WHO) and Alzheimer’s Disease International (ADI) released the report “Dementia: a public health priority" to raise awareness of dementia as an increasing threat to global health [1]. However, ignorance and misunderstanding of dementia is still common among the public in China [6]. Most people mistake cognitive dysfunction in dementia for normal process of aging, and fail to pay attention to people with dementia [14]. Even if cognitive decline cause serious consequences, for example getting lost, families are still unwilling to take people with dementia to hospitals [14]. Therapeutic nihilism of dementia is another misconception among the public. A study carried out in Shanghai showed that 45% of family members and caregivers did not believe that people with dementia could benefit from medical care [15]. Even those people who worked in mental health field believed that it was meaningless to provide people with dementia with available treatments as they may cause more harm than good [16]. Discrimination and stigmatization of dementia is another sign of poor awareness in the public. The term for dementia in Chinese is “laonian chidai”, which literally means “stupid, demented elderly”, reflecting the widely existence of misunderstanding and discrimination on dementia in China. Discrimination of people with dementia is especially common in the community of older people with low-level education. A cross-cultural investigation showed that stigmatization of dementia was higher in Chinese than in African Americans and Latinos [17]. Most families of people with dementia refused to disclose the condition to avoid stigma [18].

WHO and ADI proposed a six stage “Acceptance of Dementia” model, including: Stage 1, Ignoring the problem; Stage 2, Some awareness; Stage 3, Building dementia infrastructure; Stage 4, Advocacy efforts; Stage 5, Policies and dementia plans or strategies; and Stage 6: Normalization [19]. According to this model, China remains at the lower stages in the acceptance of dementia.

### 3.2. Inadequate Knowledge about Dementia for Medical Professionals and Caregivers

In China formal training and education about dementia are not included as part of the regular curricula in medical school [20]. As a consequence, most medical professionals lack confidence and expertise in the diagnosis, medications and non-pharmacological interventions of dementia [21]. As in Chinese PLA General Hospital, one of the best hospitals in Beijing and even in China, about 70.2% of the doctors and 46.7% of the nurses knew diagnostic procedures with respect to dementia, but only 5.0% of the doctors and 3.1% of the nurses claimed to have very good knowledge of the disease [22]. The situation is much worse in the vast rural areas of China. A survey found that in a rural county of Zhejiang province—and probably in much of rural China—all the three tiers of rural medical institutions including village clinics, township hospitals and the county hospital, lacked both clinical staff and standardized, evidence-based practices for the diagnosis, care and treatment and rehabilitation of people with dementia [23]. Inadequate knowledge about dementia for medical professionals impedes the proper diagnosis and treatment of dementia with dementia. It was estimated that only 10% of people with dementia in China were diagnosed, of which 21% were prescribed medication [24,25,26].

In China, the majority of people with dementia receive informal care at home, while only those individuals with mild symptoms and from high-income households have access to formal care at nursing homes [3]. Unfortunately, due to shortage of education programs and support services in China, most family caregivers (i.e., mainly the spouse or adult children of the people with dementia, acting as unpaid caregivers) and paid caregivers at home or nursing homes have limited knowledge and skills about dementia care [23,27]. A survey showed that a 6-week standard dementia course was available to 27 of the 31 Australian family caregivers participating in the survey, whereas most of the 68 Chinese family caregivers were unable to gain information to meet their ongoing learning needs for dementia care [28]. Another survey in eight nursing homes in Xi’an, China revealed that the caregivers showed a mastery of safety nursing, but had relatively poor knowledge about disease information, mental nursing and daily life care [29]. Inadequate knowledge about dementia for caregivers hinders the delivery of optimal care to people with dementia. Most family caregivers and nursing homes in China only provide basic care on eating, dressing and bathing, whereas specialized dementia care, such as memory/cognitive exercises and rehabilitation are still very rare in China [3].

### 3.3. Undeveloped Dementia Service System

For decades, people with dementia in China have been mainly reliant on home-based care provided by family members [28]. It was estimated that, 96% of people with dementia in China were cared at home by family members or paid caregivers [30]. According to a survey carried out in Beijing, around 45% and 1% of people with dementia paid for care in urban and rural areas, respectively [13]. The primary caregivers in the city were usually their spouses, while in rural areas the primary caregivers were sons or daughters [31]. The caregivers mainly assist with activities of daily life, such as dressing, eating, grooming and toileting. The situation whereby the majority of people with dementia are cared at home by family members could be related to three factors [28]. Firstly, traditional Chinese culture and Confucian philosophy advocate collectivism, self-sacrifice, and filial piety, making it a responsibility for family members to care the old and the sick. Secondly, the Chinese Government enforces this responsibility by law. Thirdly, nursing homes in China are not willing to admit people with dementia.

Unfortunately, the traditional family role in dementia care is weakening due to demographic shifts and socioeconomic changes. Traditional Chinese families generally consist at least two generations of people including the husband, the wife and several children. The implementation of family planning policy (also known as one-child policy) in China from 1979 to 2015 resulted in the “2:1” family structure consisting of the couple with only one child. When the only one child grows up, he or she has no brothers or sisters to share the responsibility/burden to care the old parents [32]. Meanwhile, China have millions of “empty nesters”, referring to older people living alone or with a spouse in a family without children around, making the issue of home-based care more challenging. About 31.77% older people in China were empty nesters in 2010, and the percentage is estimated to reach 90% in 2030 [33,34].

Under these new circumstances, Chinese government aims to establish three tiers of services for older people in need of care, including home-based care as the basis, community-based services as support and institutional care as a last resort. For people with dementia, both the community-based services and the institutional care are still in the developmental stages. Almost all the day-care centers for adults in China are designed to serve relatively healthy individuals and therefore lack the resources to cope with the needs of people with dementia [23]. The availability of community-based dementia-specific services, such as day care, respite, caregiver support and case management, is still very limited, except in very few major urban centers like Hong Kong and Shanghai [35,36].

With the government’s policy inducement on institutional care, the number of institutional care facilities in China rapidly increased to 40,000 (with 3.15 million beds) in 2010 [35]. However, the quality of institutional care for people with dementia is problematic. Institutional care facilities rarely have clinical staff, and most of employees lack training and education on management of dementia [37,38]. As a consequence, many institutions simply refuse to admit people with dementia [38]. A survey in a rural county of Zhejiang province found that none of the people with dementia in nursing homes received dementia-related medication, psychological care or rehabilitative support [23].

### 3.4. High Cost of Dementia Care

Dementia care imposes significant economic burden on people with dementia and their families in China. A study performed in Shanghai, the largest city in China showed that the average direct cost per person with dementia per year was approximately 8432 RMB (i.e. Chinese Yuan, 1 RMB = 0.15 USD), and the indirect cost was 10,568 RMB [39]. Another study showed that the average total economic loss of a person with dementia was 1160 RMB per month, including 712 RMB per month for health costs. Based on the data, in 2009 the calculated annual total economic loss in China was between 83.5 and 97.4 billion RMB and the annual health care cost for dementia was 51.3–59.8 billion RMB [7]. The impact of Alzheimer-type dementia on China’s economy was estimated to exceed $1 trillion USD in 2050 [40]. Considering that AD accounts for 50%–75% of total dementia cases, other types of dementia including VaD, FTD and DLB would have significant economic impacts on China as well.

Due to the marked urban bias of public policy, older people living in rural China have much lower incomes and lack amenities, and thus have lower utilization of healthcare resources [13]. A survey about the prevalence and care of chronic diseases (including dementia) showed that in rural areas only 6.1% of older people with chronic disease were likely to use health services in a three-month period, as opposed to 38.6% of urban residents [13]. Another survey on long-term care revealed that only 5.9% of the disabled older people in rural area received any formal care, whereas the rate in urban area was 36.9% [31]. A Delphi panel study about Alzheimer-type dementia in urban Shanghai found that the presence of comorbidities, symptoms of agitation/aggression, and loss of functional independence at the moderate to severe stage of the disease were the driving factors for healthcare resource utilization at hospitals or nursing homes [3]: (i) dependent people with dementia required 12–15 h/day of caregiver time, while independent individuals required 1–3 h/day; (ii) between 70% and 90% of dependent/aggressive people with dementia were able to be hospitalized, while up to 20% were able to be to be accepted in a nursing home; (iii) dependent/non-aggressive people with dementia had 5%–35% probability of hospitalization and an 80% probability of being accepted in a nursing home. The majority of healthcare cost was attributed to hospitalization (30,000 RMB/month) and formal care-giving (6000 RMB/month), provided either in a nursing home or by home-based professional caregivers [3].

The cost of services for dementia care is covered by health insurance and out-of-pocket payments [36]. In China, there are two urban insurance schemes either for those in formal employment, or for those who are not employed or in informal employment, and one rural insurance scheme which has much lower reimbursement levels and service coverage than the urban ones [41]. There is also the Medical Assistance Program (MA) to help people who cannot afford health insurance premiums [42]. Although the breadth of healthcare insurance coverage in China reached 95% of its entire population in 2011, its comprehensiveness, quality and capacity of financial protection remain insufficient [42]. It was reported that the medications used for dementia treatment were relatively expensive and not always covered by health insurance [23], and most long-term care was paid for out-of-pocket [43]. A survey in 2009 revealed the monthly out-of-pocket cost associated with dementia care in China [44]. The total out-of-pocket monthly direct cost per person was US$487, including US$56 for doctor visits, US$137 for drug therapy, US$139 for hospital visits, and US$155 for a hired housekeeper. As for the total out-of-pocket monthly indirect costs (i.e., unpaid care provided by family members and others) per person, this was estimated to range from US$88 to US$614. The average total monthly out-of-pocket cost of dementia care far exceeded national average monthly incomes (US$210 for urban residents and US$63 for rural residents) [45]. Thus, for people with dementia in China, the high cost of dementia care not only restrains utilization of healthcare services, and also increases the risk of disease-induced impoverishment.

## 4. Strategies to Improve Dementia Care in China

China has the largest number of people with dementia worldwide, but lacks a mature service system to cope with the enormous challenges of dementia care. ADI proposed a dementia care services model for the middle-income and low-income countries. The model emphasized to promote public awareness and understanding of dementia as the first step to reduce the risk factor for dementia and gradually develop dementia care services through primary health care and then the senior one [2]. The achievement of the Alzheimer’s plan of action (APA) of Mexico showed that the ADI model was applicable in middle-income and low-income countries. The APA was implemented in 2014, aiming to increase social awareness, eliminate the stigma of dementia and increase the training of health professionals and caregivers [6]. The plan has made much progress in promoting the social awareness and training of health professionals by three training strategies which would be of reference value for China. The training strategies included a 160-h dementia course with diploma and guided by experts, an online course for health professionals, and an open online course through the Tele-education platform. The next challenges for Mexico are to strengthen the primary care system and support caregivers. The Indonesian Ministry of Health also launched a dementia plan in March 2016 which makes awareness promotion the primary target [6].

The ADI model is applicable for China, and China is taking action to address the challenges of dementia care. Although there is no specific National Dementia Plan, China has launched the National Five-year Plan for Mental Health (with dementia prioritized) in 2015 [46]. The plan aims to promote the social awareness through mental health propaganda and education in hospitals, schools and communities, and to train more mental health specialists with no less than 40,000 registered mental doctors. Meanwhile, it targets to develop an integrated mental health service system, including mental health professional institutions, rehabilitation services and community- and family-based supports by 2020 [46].

On the other hand, the 13th Five-year Development Plan (2016–2020) will deepen the reform of health service system of China [47]. One emphasis of the health reform is to strengthen the three-tiered healthcare service system, in particular, to shift the medical resources towards the primary level and promote greater care integration between hospitals and primary care facilities. The priority task in developing primary care is to train a sufficient, qualified primary health care (PHC) workforce (i.e., two to three licensed primary care doctors per 10,000 people by 2020) [48]. To achieve this goal, China will promote the status of the general practice specialty, establish independent professional licensing and career development system, and construct compensation system for the PHC workforce. Another emphasis of the health reform is to support “people first principles” such as building harmonious relationships with patients [47]. The improvement of primary care and a patient friendly service system will lead to better outcomes for people with dementia

The reform of health insurance is in progress as well. The government aims to establish a consolidated health insurance system by 2020. As piloted in several cities, the consolidated health insurance system increased the reimbursement for inpatient services and decreased total expenditures for both outpatient and inpatient services [41]. This will probably increase the affordability of healthcare resource utilization and provide greater social support for people with dementia.

## 5. Conclusions

In summary, providing care to nearly 10 million people with dementia is an unprecedented challenge for China. There is urgent need to increase dementia awareness of the public, to educate and train health professionals and caregivers, and to develop policy, service and resources for dementia care. Although the improvement of dementia care is a long-term and arduous task, eventually it will benefit millions of older people in China and promote the harmonious development of the society.

## References

[1] Dementia: A Public Health Priority. http://www.who.int/mental_health/publications/dementia_report_2012/en/.

[2] World Alzheimer Report 2009: The Global Prevalence of Dementia. http://www.alz.co.uk/research/world-report-2009.

[3] Yu X., Chen S., Chen X., Jia J., Li C., Liu C., Toumi M., Milea D. (2015). Clinical management and associated costs for moderate and severe Alzheimer’s disease in urban China: A Delphi panel study. Transl. Neurodegener..

[4] Boustani M., Schubert C., Sennour Y. (2007). The challenge of supporting care for dementia in primary care. Clin. Interv. Aging.

[5] World Alzheimer Report 2010: The Global Economic Impact of Dementia. http://www.alz.co.uk/research/world-report-2010.

[6] World Alzheimer Report 2016: Improving Healthcare for People Living with Dementia. https://www.alz.co.uk/research/world-report-2016.

[7] Song Y., Wang J. (2010). Overview of Chinese research on senile dementia in mainland China. Ageing Res. Rev..

[8] Wu Y.T., Lee H.Y., Norton S., Chen C., Chen H., He C., Fleming J., Matthews F.E., Brayne C. (2013). Prevalence studies of dementia in mainland china, Hong Kong and Taiwan: A systematic review and meta-analysis. PLoS ONE.

[9] Guo F., Zhang Z.X. (2007). Epidemiological research status of dementia. Chin. J. Neurol..

[10] Tang Z., Meng C., Dong H.Q., Wu X.G., Min B.Q. (2002). Studies on the incidence of senile dementia in urban and rural areas of Beijing City. Chin. J. Gerontol..

[11] Wu Y.T., Lee H.Y., Norton S., Prina A.M., Fleming J., Matthews F.E., Brayne C. (2014). Period, birth cohort and prevalence of dementia in Mainland China, Hong Kong and Taiwan: A meta-analysis. Int. J. Geriatr. Psychiatry.

[12] Wu Y.T., Grant W.B., Prina A.M., Lee H.Y., Brayne C. (2015). Nutrition and the prevalence of dementia in mainland China, Hong Kong, and Taiwan: An ecological study. J. Alzheimers Dis..

[13] Liu Z., Albanese E., Li S., Huang Y., Ferri C.P., Yan F., Sousa R., Dang W., Prince M. (2009). Chronic disease prevalence and care among the elderly in Urban and Rural Beijing, China—A 10/66 Dementia Research Group cross-sectional survey. BMC Public Health.

[14] Dai B., Mao Z., Mei J., Levkoff S., Wang H., Pacheco M., Wu B. (2013). Caregivers in China: Knowledge of mild cognitive impairment. PLoS ONE.

[15] Li X., Fang W., Su N., Liu Y., Xiao S., Xiao Z. (2011). Survey in Shanghai communities: The public awareness of and attitude towards dementia. Psychogeriatrics.

[16] Hsiao H.Y., Liu Z., Xu L., Huang Y., Chi I. (2015). Knowledge, Attitudes, and clinical practices for patients with dementia among mental health providers in China: City and town differences. Gerontol. Geriatr. Educ..

[17] Mahoney D.F., Cloutterbuck J., Neary S., Zhan L. (2005). African American, Chinese, and Latino family caregivers’ impressions of the onset and diagnosis of dementia: Cross-cultural similarities and differences. Gerontologist.

[18] Sun F. (2014). Caregiving stress and coping: A thematic analysis of Chinese family caregivers of persons with dementia. Dementia (Lond.).

[19] Lin S.Y., Lewis F.M. (2015). Dementia friendly, dementia capable, and dementia positive: Concepts to prepare for the future. Gerontologist.

[20] Country’s Mental Health Services Lacking. http://www.chinadaily.com.cn/cndy/2012-05/16/content_15302489.htm.

[21] Liu J.Y., Lai C., Dai D., Ting S., Choi K. (2013). Attitudes in the management of patients with dementia: Comparison in doctors with and without special training. East Asian Arch. Psychiatry.

[22] Jiang R., Shan X., Zhang R., Wang X. (2009). Comparison between doctors and nurses cognition about the senile dementia. Chin. J. Clin. Healthc..

[23] Wu C., Gao L., Chen S., Dong H. (2016). Care services for elderly people with dementia in rural China: A case study. Bull. World Health Organ..

[24] China Alzheimer’s Project—Memory360. http://www.memory360.org/en/#1.

[25] Chen R., Hu Z., Chen R.L., Ma Y., Zhang D., Wilson K. (2013). Determinants for undetected dementia and late-life depression. Br. J. Psychiatry.

[26] Chen S., Boyle L.L., Conwell Y., Chiu H., Li L., Xiao S. (2013). Dementia care in rural China. Ment. Health Fam. Med..

[27] Wang J., Xiao L.D., Li X., de Bellis A., Ullah S. (2015). Caregiver distress and associated factors in dementia care in the community setting in China. Geriatr. Nurs..

[28] Xiao L.D., Wang J., He G.P., De Bellis A., Verbeeck J., Kyriazopoulos H. (2014). Family caregiver challenges in dementia care in Australia and China: A critical perspective. BMC Geriatr..

[29] Cui Y., Fan R., Wang Y.M., Kaye A.J., Kaye A.D., Bueno F.R., Pei J.M. (2014). A changing healthcare system model: The effectiveness of knowledge, attitude, and skill of nursing assistants who attend senile dementia patients in nursing homes in Xi’an, China—A Questionnaire Survey. Ochsner J..

[30] Zhang Z.X., Chen X., Liu X.H., Tang M.N., Zhao H.H., Jue Q.M., Wu C.B., Hong Z., Zhou B. (2004). A caregiver survey in Beijing, Xi’an, Shanghai and Chengdu: Health services status for the elderly with dementia. Acta Acad. Med. Sin..

[31] Li M., Zhang Y., Zhang Z., Zhang Y., Zhou L., Chen K. (2013). Rural-urban differences in the long-term care of the disabled elderly in China. PLoS ONE.

[32] Flaherty J.H., Liu M.L., Ding L., Dong B., Ding Q., Li X., Xiao S. (2007). China: The aging giant. J. Am. Geriatr. Soc..

[33] Su D., Wu X.N., Zhang Y.X., Li H.P., Wang W.L., Zhang J.P., Zhou L.S. (2012). Depression and social support between China’ rural and urban empty-nest elderly. Arch. Gerontol. Geriatr..

[34] Zhang Y. (2013). The home resident, health and care arrangements of the aging population in China: The sixth census data analysis. Jiangsu Soc. Sci..

[35] Feng Z., Liu C., Guan X., Mor V. (2012). China’s rapidly aging population creates policy challenges in shaping a viable long-term care system. Health Aff. (Millwood).

[36] Gallagher-Thompson D., Tzuang Y., Au A., Wang D., Tsien T., Wang P., Catholic F., Huang Y., Karen E., Shripad T. (2010). Families dealing with dementia: Insights from China, Hong Kong, and Taiwan. Aging Asia: The Economic and Social Implications of Rapid Demographic Change in China, Japan, and South Korea.

[37] Chen S., Boyle L.L., Conwell Y., Xiao S., Chiu H.F. (2014). The challenges of dementia care in rural China. Int. Psychogeriatr..

[38] Wu S., Dong B., Ding G., Chen J., Pang W. (2011). Status quo of reception and care for patients with senile dementia in pension agencies in Chengdu. Mod. Prev. Med..

[39] Wang G., Cheng Q., Zhang S., Bai L., Zeng J., Cui P.J., Zhang T., Sun Z.K., Ren R.J., Deng Y.L. (2008). Economic impact of dementia in developing countries: An evaluation of Alzheimer-type dementia in Shanghai, China. J. Alzheimers Dis..

[40] Keogh-Brown M.R., Jensen H.T., Arrighi H.M., Smith R.D. (2016). The impact of Alzheimer’s disease on the Chinese economy. EBioMedicine.

[41] Meng Q., Fang H., Liu X., Yuan B., Xu J. (2015). Consolidating the social health insurance schemes in China: Towards an equitable and efficient health system. Lancet.

[42] The Long March to Universal Coverage: Lessons from China. https://openknowledge.worldbank.org/handle/10986/13303.

[43] Feng Z., Zhan H.J., Feng X., Liu C., Sun M., Mor V. (2011). An industry in the making: The emergence of institutional elder care in Urban China. J. Am. Geriatr. Soc..

[44] Mould-Quevedo J.F., Tang B., Harary E., Kurzman R., Pan S., Yang J., Qiao J. (2013). The burden of caring for dementia patients: Caregiver reports from a cross-sectional hospital-based study in China. Expert Rev. Pharmacoecon. Outcomes Res..

[45] China Statistical Yearbook 2010. http://www.stats.gov.cn/tjsj/ndsj/2010/indexeh.htm.

[46] The National Five-Year Plan for Mental Health (2015–2020). http://www.gov.cn/zhengce/content/2015-06/18/content_9860.htm.

[47] Deepening Health Reform in China: Building High-Quality and Value-Based Service Delivery. http://www.indiaenvironmentportal.org.in/content/433080/deepening-health-reform-in-china-building-high-quality-and-value-based-service-delivery/.

[48] Wu D., Lam T.P. (2016). Underuse of primary care in China: The scale, causes, and solutions. J. Am. Board Fam. Med..

